# Multielement Determination in Turmeric (*Curcuma longa* L.) Using Different Digestion Methods

**DOI:** 10.3390/molecules27238392

**Published:** 2022-12-01

**Authors:** Michaela Zeiner, Monika Šoltić, Ivan Nemet, Iva Juranović Cindrić

**Affiliations:** 1 Man-Technology-Environment Research Centre, School of Science and Technology, Örebro University, Fakultetsgatan 1, 70182 Örebro, Sweden; 2 Department of Chemistry, Faculty of Science, University of Zagreb, Horvatovac 102a, 10000 Zagreb, Croatia

**Keywords:** atomic spectrometry, *Curcuma longa* L., elemental pattern, macro- and micro elements, microwave-assisted sample preparation

## Abstract

The antioxidant, anti-inflammatory and antiseptic properties of turmeric (*Curcuma longa* L.) derive from its rich nutritional composition making it interesting for medicinal uses, besides being used as spice in cooking. To complete the picture on the composition of turmeric, not only the organic compounds need to be known, but also the elemental composition covering essential and potentially toxic elements. The samples were digested in a microwave assisted digestion system using different reagent mixtures. The best digestion mixture was semi-concentrated nitric acid combined with hydrogen peroxide. After optimization of the sample preparation method, the contents of Ag, Al, As, Ba, Be, Bi, Ca, Cd, Co, Cr, Cu, Fe, Ga, K, Li, Mg, Mn, Mo, Na, Ni, Pb, Rb, Se, Sr, Te, Tl, V and Zn in curcuma were determined by inductively coupled plasma atomic emission spectrometry (ICP-AES), as well as by inductively coupled plasma mass spectrometry (ICP-MS). Even if the general composition found is in line with the scarce data in literature, clear differences can be seen between the analyzed samples, considering provenience, production procedures, and harvesting year as potential influencing factors. Whereas all samples contained less As and Pb than regulated by WHO, one limit exceeding was found for Cd.

## 1. Introduction

Turmeric (*Curcuma longa* L.) is a plant from the ginger family (*Zingiberaceae*), and because of the yellow color of the stem, it is also known as Indian saffron [1]. The rhizome, from which turmeric powder is obtained by drying and grinding, is yellowish-brown on the outside, while the inside is intensely yellow-orange. Turmeric rhizome is extremely aromatic and has a characteristic smell, a slightly bitter taste and is rich in numerous active ingredients [2]. The geographical conditions of the growing area and the characteristics of the soil can significantly affect the growth and elemental composition of the plant [1], so that samples from various origins have to be investigated.

Turmeric is traditionally used for its antioxidant, antiinflammatory, antimicrobial, anticancer and many other beneficial properties. Due to its antioxidant effect, it is often used in cosmetics in creams, serums and other products to reduce skin aging problems. The composition of the bioactive compounds has already been widely studied [1,3,4,5]. The medicinal properties of turmeric have been known since ancient times, but the exact mechanisms of action and the determination of biologically active components have only recently been investigated. [5,6] The production of these bioactive compounds, the secondary metabolites, is related to various stress factors, such as heavy metal contamination of the growing site [7]. These alterations in plant physiology can also alter the potency of medicinal plants [8]. The determination of metals and metalloids in plants used for medicinal and or nutritional purposes is important, not only regarding the nutritional information by essential elements, but also potentially toxic ones have to be studied and quantified. The World Health Organization (WHO) limits only the contents of As, Cd and Pb, in raw plant materials, namely with the following permissible values 1.0 mg/kg, 0.3 mg/kg and 10 mg/kg, respectively [9]. Especially since metal pollution in the environment is an increasingly serious problem, the elemental characterization of commonly used medicinal plants is of importance. Plants take up metals and metalloids via roots and leaves [10], and resultant accumulation of potentially toxic metals can consequently affect human physiological functions through the food chain [11]. Only a few publications dealing with different metals and metalloids determined in turmeric are available [3,12,13,14]. The place of growing, the type of cultivar, the further processing and the time of harvest are important influencing parameters for the final elemental composition of a certain plant, but are mainly not mentioned in the respective studies, probably due to secrecy by the selling companies. The aim of the present study is the multielement analysis of turmeric samples commercially available on the Croatian market, all originating from India. Furthermore, the optimization of the sample preparation was intended. Elemental analysis of a sample often requires preliminary preparation. It is necessary to disrupt the sample, degrade the matrix and release the analyte to be in a suitable form for analysis. Given that biological samples consist of a complex organic matrix (a mixture of carbohydrates, proteins and lipids), the sample must be decomposed, which converts the elements bound in the organic material into an inorganic form, and then into an aqueous solution that is used in further analytical procedures [15] This step of the entire analytical procedure is not only time- and labor-intensive, but also needs various reagents, which results in a significant contribution of overall uncertainty of the analytical procedure used [16,17]. Reducing the number of different reagents, as well as their volumes needed, is also of concern regarding green analytical chemistry [18,19].

## 2. Results and Discussion

### 2.1. Methodology

#### 2.1.1. Sample Preparation

Elemental analysis of the turmeric samples by both inductively coupled plasma atomic emission spectrometry (ICP-AES) and inductively coupled plasma mass spectrometry (ICP-MS) was performed after a preceding microwave-assisted digestion step. In this work, nitric acid and a mixture of nitric acid and hydrochloric acid, with and without the addition of hydrogen peroxide, were used to decompose the sample matrix. Since the degradation of the sample is not only affected by the choice of reagents, but also by the relative proportions of each reagent, the digestion was also carried out using different volume ratios of the respective reagents. Five different digestion mixtures (A–E) were tried to find the optimal conditions for the matrix of interest, i.e., turmeric in powdered form. (A: 3 mL HNO_3_ conc, B: 6 mL HNO_3_ (50:50 *v*/*v*) + 2 mL H_2_O_2_ (1 mol L^−1^), C: 1 mL HNO_3_ conc + 4 mL H_2_O + 2 mL H_2_O_2_ (1 mol L^−1^), D: 6 mL HNO_3_ conc + 2 mL H_2_O_2_ (1 mol L^−1^) and E: 1 mL HNO_3_ conc + 3 mL HCl conc).

**Figure 1:**
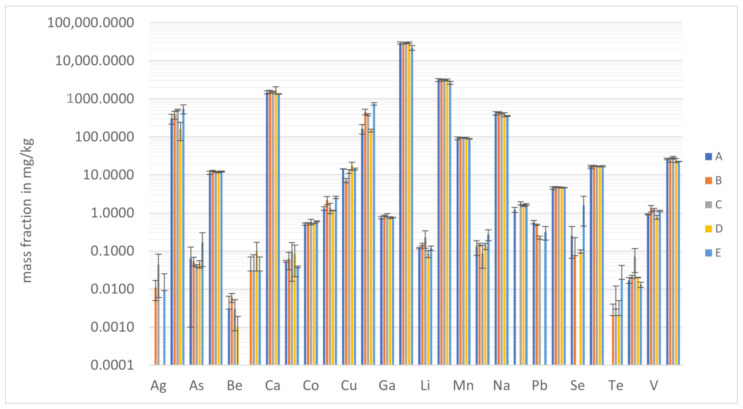
Mass fraction of all analytes in mg/kg obtained for sample K1 after digestions A–E. Error bars refer to ±SD.

Figure 1 shows the obtained results for all analytes in sample K1 by the five different digestion methods (also presented in Table S1). Using only concentrated nitric acid, incomplete digestion was observed. Prior to introducing these solutions into the analytical instruments, a filtration step was necessary. Apart from the higher workload, this leads to a potential loss of analytes [20]. Conversely applying the other four digestion mixtures, clear digest solutions were obtained. The results for each method were pairwise checked for statistically significant differences using a dependent sample Student’s *t*-test. The assumption that there is no significant difference between the obtained data sets was tested at the significance level of 0.01. No statistically significant differences were found between the digestion mixtures B to E, thus all mixtures can be applied in order to get reliable data. Hydrochloric acid in the digestion mixture leads to high chloride concentrations in the digest solution, so that polyatomic interferences are to be expected when using ICP-MS, especially for arsenic (from ^40^Ar^35^Cl^+^) and vanadium (from ^35^Cl^16^O^+^) [21] thus, reagent mixture E was not further taken into consideration. C and D were not chosen for the final method, in order to avoid handling concentrated acids. Thus, mixture B was selected for the further investigation. The chosen sample pretreatment procedure is characterized by ease of use, minimization of contamination and experimental errors by reducing the number of different reagents and reducing interferences in the following measurements.

#### 2.1.2. Quantification of the Elements by Atomic Spectrometry

For method validation, precision and trueness were determined by analyzing four different certified reference materials (CRM) in plant matrix (peach leaves, tomato leaves, strawberry leaves, algae). The obtained results show that the chosen method of microwave-assisted digestion using reagent mixture B gives valid results of elemental analysis and proves the choice of this sample preparation procedure with precision data ranging from <1% to 4% and recoveries for the certified elements from 82% to 115% for both methods (ICP-AES, ICP-MS). The latter also confirm the usage of external calibration, all curves having coefficients of determination R^2^ beyond 0.997. The LODs for all elements in the samples were determined to be below 3 mg/kg as found in previous studies [22]. The sensitivity, i.e., the slope of the calibration curves, alongside the intercepts of the trendline equations for all analytes, and all LODs are listed in Table S2.

### 2.2. Elemental Composition of Turmeric Samples

**Figure 2:**
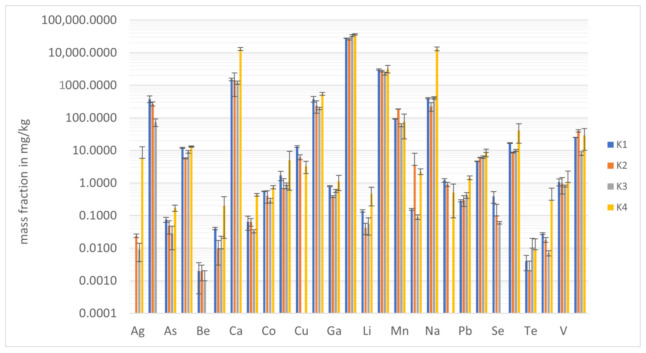
Elemental mass fractions (in mg/kg) for the analyzed samples of turmeric. Error bars refer to ±SD.

The digest solutions of all turmeric samples were measured by two methods for elemental analysis (ICP-MS and ICP-AES). For most elements, similar mass concentrations were obtained using both methods, and then their mean values were further treated. Elements present in very low concentrations in the obtained solutions were only quantified by ICP MS. The summary of the data is presented in Figure 2. For better clarity, all values are also given in Table S3.

Four publications containing mass fraction data for several of the investigated elements will be used in the further discussion. Li et al. [3] quantified nine elements, As, Hg, and Pb by GF-AAS (graphite furnace atomic absorption spectrometry) alongside Ag, Ba, Cd, Cr, Se, and Ni by ICP-AES, sample preparation and provenience of samples not being described in the paper. Maghrabi reported the mass fraction of 18 metals and metalloids in Indian turmeric samples determined by ICP-AES after acidic microwave assisted digestion. The paper covers trace elements, micro- and macronutrients, namely Al, Ba, Ca, Cd, Co, Cr, Cu, Fe, K, Mg, Mn, Na, Ni, Pb, Sb, Se, V, and Zn [12]. Muralidharan and coworkers analysed Indian turmeric for the content of eleven elements, i.e., As, Ca, Cd, Cr, Cu, Fe, Hg, Mg, Mn, Pb, and Zn, by ICP-AES. They performed open vessel digestion using perchloric and nitric acid [13]. The study by Silva and colleagues focused as the presented one on a wide range of elements, which were determined by three different methods, namely As, Ba, Br, Ca, Cl, Co, Cr, Cs, Fe, Hf, K, Na, Mg, Mn, Rb, Sb, Sc, Se, Ti, V and Zn, by instrumental neutron activation analysis (INAA); Cd, Cu, Ni and Pb by ICP-AES) and Hg by cold vapor atomic absorption spectrometry (CV AAS). Their samples purchased in dried form underwent microwave assisted digestion using a mixture of four reagents prior to analysis by the latter two methods. The origin of the samples is not given [14]. Based on the scarce literature data, comparison and general conclusion cannot be easily drawn regarding influencing parameters, such as sample provenience, processing, harvesting year, alongside climatic conditions.

Regarding the macro-elements essential for humans, Ca, K, Mg, and Na were found in all analysed samples. The most abundant element in all samples (K1–K4) is K in a content range of 26.3 g/kg–36.2 g/kg, followed by Mg with a value range of 2.29 g/kg–3.20 g/kg, Ca (1.20 g/kg–1.52 g/kg, K4 excluded) and Na (223 mg/kg–408 mg/kg, K4 excluded). Only in sample K4, higher contents were found for Ca and Na, namely 12.9 g/kg and 13.1 g/kg, respectively. Na is an indicator for soil salinity which was found to alter to uptake of metals by plants [23], thus the growing area alongside the respective climatic conditions seem to influence the value found in this sample. Maghrabi determined a content of 24.1 g/kg for K [12], while Silva et al. determined a little lower content, namely 19.9 g/kg K [14], which is in accordance with the obtained results. The mass content of Ca in turmeric samples is in a comparable range compared to the literature data (1.20 g/kg, 1.77 g/kg, and 1.85 g/kg, resp.) [12,13,14] except for K4 with an almost 10-times higher value.

The mass fractions determined for Mg is comparable to the findings by by Maghrabi (2.06 g/kg, [12]) and Muralidharan and coworkers (2.32 g/kg, [13]), but twice as high as the value obtained by Silva et al. (1.20 g/kg, [14]).

Literature data show significantly lower values for Na (133 mg/kg [12], 76 mg/kg [14]) than the obtained results.

All microelements could be found in sample K2, whilst single elements were below LOD in the other samples, namely Ag in K1, Cu and Ni in K3, and Al, Be, and Se in K4. A summary of the obtained mass fractions is presented in Figure 2.

The Al contents found in K1 to K3 ranged from 73 mg/kg–379 mg/kg, whilst a higher value was determined by Maghrabi (732 mg/kg) [12].

In contrast to Al, the results for Fe and Mn are closer to the published data. Samples K1 to K4 contained Fe in the range of 192 mg/kg–542 mg/kg and Mn in the range of 59.1 mg/kg–185 mg/kg: Silva and coworkers founds around 327 mg/kg for Fe and 193 mg/kg for Mn [14], while Maghrabi determined slightly higher values for both, namely for Fe 726 mg/kg and 571 mg/kg for Mn [12]. Muralidharan and coworkers however had a similar result for Fe (177 mg/kg), but a much lower one for Mn (34.1 mg/kg) [13].

A closer look on the other microelements found in turmeric reveals, in decreasing order of mass fraction, the following group Ba, Cu, Rb, Sr, and Zn. Cu and Zn, which are considered essential microelements necessary for the normal functioning of numerous enzymes, were found in the range <LOD–13.1 mg/kg and 8.00 mg/kg–39.7 mg/kg, respectively. These data are in accordance with those reported in literature (6.01 mg/kg and 37.3 mg/kg [12]; 7.14 mg/kg and 15.8 mg/kg [14]). Muralidharan et al. did not determine Cu, but for Zn their results are also in the similar range (13.8 mg/kg–28.1 mg/kg) [13].

Ba has no essential role for human health, and some of its compounds might even be toxic to humans. The analyzed samples contained between 5.6 mg/kg and 13.1 mg/kg Ba, the literature data covering a wide range, i.e., 13.3 mg/kg [3], 20 mg/kg [14], 60.4 mg/kg [12]. The monovalent cation Rb has be found in the range from 4.6 mg/kg up to 8.6 mg/kg, which is lower than reported by Silva and colleagues [14].

Strontium occurs naturally in the soil and is transferred to plants via roots. Due to its similarity to Ca, elevated Sr uptake can cause Ca deficiencies [24]. The analyzed samples contained 8.6 mg/kg up to 41 mg/kg Sr, whereby the highest content was found in K4.

Ag has no important function in the human body and is present in turmeric only in very small amounts. In K1 the content is even below the LOD (0.05 mg/kg), in K2 and K3 0.02 mg/kg and 0.01 mg/kg were found, respectively. As already seen for other elements K4 is an exception also regarding Ag, containing even 6.0 mg/kg. Li et al. could not detect Ag in their sample either, their LOD being 0.4 mg/kg [3].

Cr is an essential microelement for both plants and humans. Whilst K1 to K3 contained 0.66 mg/kg to 1.7 mg/kg, the highest content was registered for sample being 5.0 mg/kg. The literature data reported for Cr in turmeric are similar to K1 to K3, namely 1.0 mg/kg [14], 2.68 mg/kg [12] or even <LOD (0.22 mg/kg) [3].

One element that causes numerous negative effects on organs and functions in the body is Pb [25]. Li et al. obtained a similar result for this metal (0.22 mg/kg) as found in K1 to K3. Exception is sample K4 with a mass fraction of 1.44 mg/kg Pb. Nevertheless, all registered values are significantly lower than the highest permissible mass fraction in medicinal plants according to the WHO, which is stated to be 10 mg/kg [9].

Cadmium is another metal, that is known for causing harmful effects in humans [26], thus its maximum content in medicinal plants is regulated by the WHO [9]. Sample K4, however, contained 0.435 mg/kg Cd, which is above the maximum permissible content (0.3 mg/kg, [9]). A similar value for Cd was determined by Maghrabi (0.36 mg/kg, [12]). Considering the amounts of turmeric used for cooking or medicinal purposes and the fact that Cd has a low bioavailability [27], the present mass content of Cd determined in K4 is not supposed to pose any danger to human health. The other turmeric samples contain Cd in the range of 0.033 mg/kg to 0.065 mg/kg and in line with the result obtained by Silva et al., namely 0.02 mg/kg–0.04 mg/kg [14]. These values are significantly lower than the maximum permissible mass content of Cd in medicinal plants.

The higher mass fractions of potentially toxic elements in K4 might be an indication of a growing area with elevated soil salinity. The elevated accumulation of harmful elements in the roots was found for plants grown under salt stress [23].

**Figure 3:**
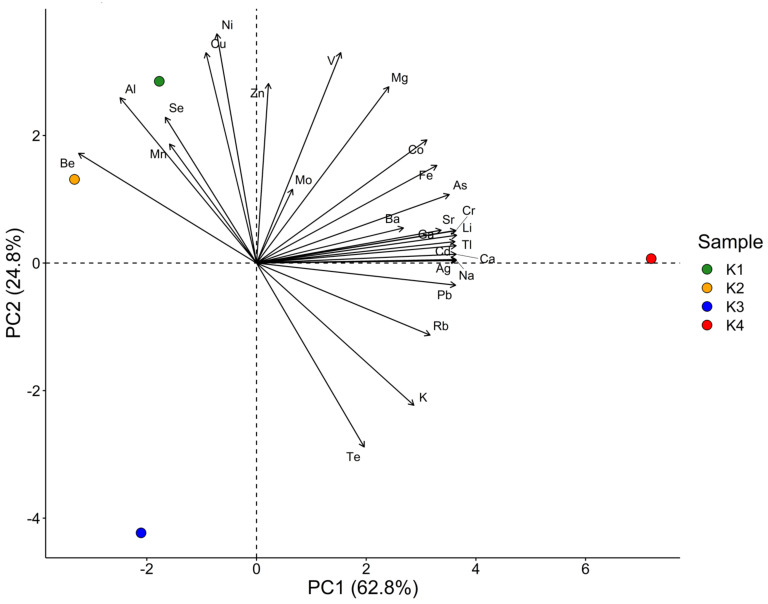
PCA-Biplot for the four turmeric samples considering all analytes.

**Figure 4:**
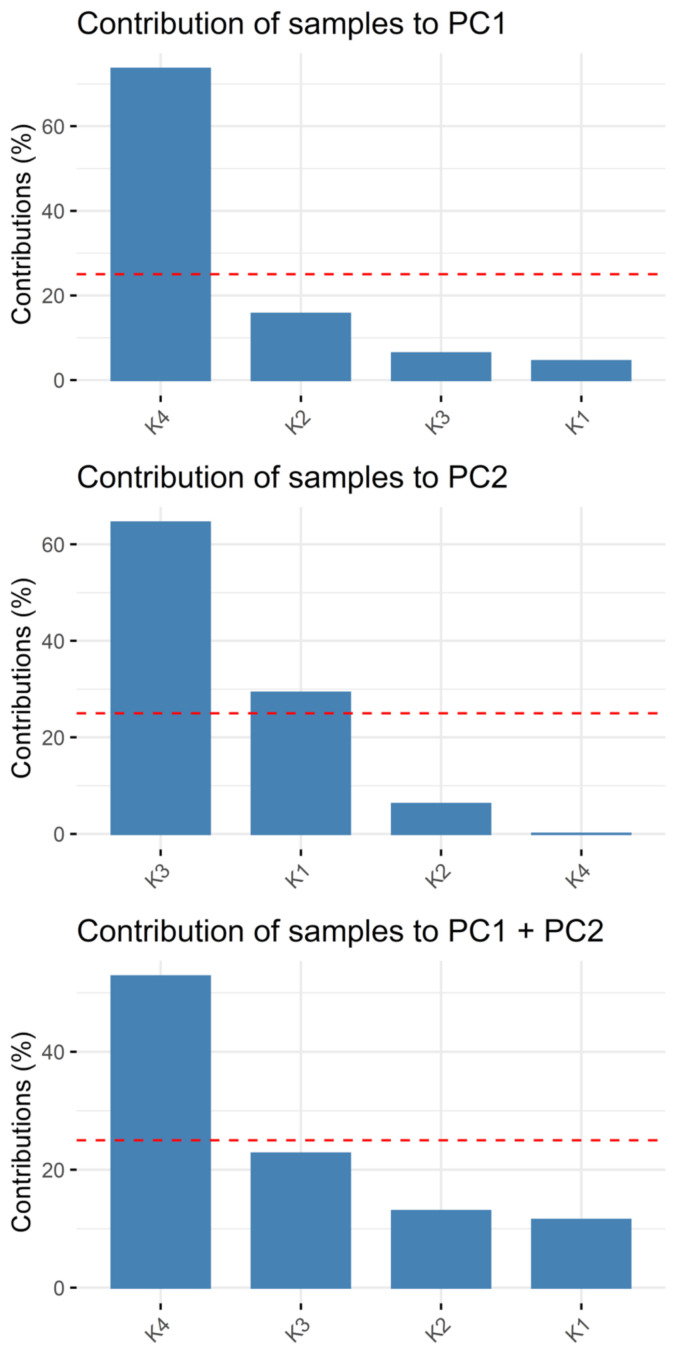
Contribution of samples to PC1 and PC2, red dashed line indicating average percentage.

**Figure 5:**
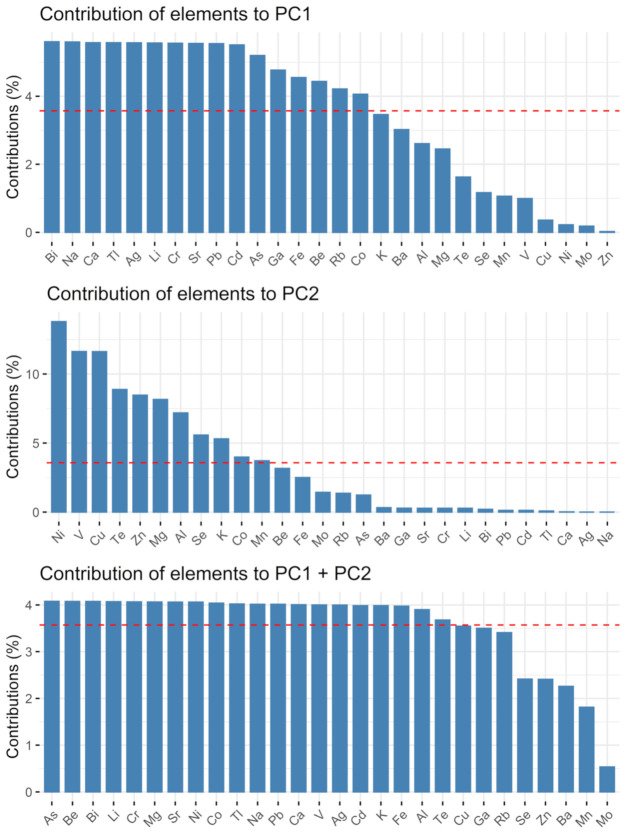
Contribution of elements to PC1 and PC2, red dashed line indicating average percentage.

The contents obtained for all quantified macro- and microelements in the turmeric samples underwent a PCA. The main results are visualized by the biplot PC1 vs. PC2, depicted in Figure 3, followed by Figure 4 and Figure 5 showing the single contributions of either the individual sample or the analytes to PC1 and PC2. No other PCs are considered since almost 90% of the variation within the dataset can be described by PC1 and PC2. Looking at PC1, the closeness of the arrows on the right-hand side highlights the positive correlation of many analytes, whilst they are negatively correlated to Be, Al, Se, and Mn, in decreasing order. Especially Ag, Na, Ca, Cd, Tl, Cr, Li, Ga, and Sr contribute almost equally to PC1 (see Figure 5). PC2 is dominated by Ni, V, Cu, and Zn, in contrast with Te and K. As already seen before, K1 and K2 are the most similar to each other, whilst K3 and K4 differ significantly. In particular, sample K4 is characterized by high values of all the many elements grouped in the close arrows pointing to high values of PC1, i.e., approximately between Mg and K, and by low values of Be, Al, Se, Mn. Samples K1 and K2 present high values of Be, Al, Mn, Se, Cu, Ni, and, less for K2, Zn, while they show low values particularly for Te, K, Rb, Pb, and all the others grouped right of PC1. Finally, K3 is characterized by low values of V, Mo, Mg, Co, and medium-low values of the two groups mentioned for K1 and K2. K4 dominates PC1, which can be clearly seen in Figure 4, its column being the only one much above the average contribution percentage indicated by the red dashed line. Conversely, PC2 is dominated by K3, followed by K1 (see Figure 4). Regarding the contribution of the single analytes to PC1, the high percentage of Na and Ca (see Figure 5) is based on the difference between K4 and the other three samples. In contrast to the quite equal total contribution of 16 elements to PC1 and PC2 (As, Be, Bi, Li, Cr, Mg, Sr, Ni, Co, Tl, Na, Pb, Ca, V, Ag, and Cd), Mo is the only element having a far lower total contribution to PC1 and PC2 than the other elements. The known origin of all samples is India, but no further information on growing area or cultivar is available due to secrecy by the companies. Thus, it can only be speculated that soil salinity might be one reason for the elevated sodium level. The potentially toxic elements with significant influence on the variation within the dataset might be an indicator for local pollution of the soil in the respective growing area. A contamination during handling is less likely for PTEs than for Fe alongside its alloying elements as steel is part of many containers and grinding devices in food processing.

## 3. Materials and Methods

### 3.1. Samples

The present study is based on four commercially available samples of turmeric were. All samples were purchased in different supermarkets in Zagreb, Croatia, in 2020. The growing country was in all cases India, whilst the further processing was performed in different (European) countries. Samples K1, K3 and K4 were sold in ground form, whilst K2 was finely powdered. So, neither grinding nor drying was necessary prior to sample digestion for all samples studied.

### 3.2. Sample Preparation

To reduce potential contamination, all glass- and plastic-ware were pre-cleaned with semi-concentrated nitric acid prior to use. Ultra-pure water (18.2 MΩ) and supra-pure reagents (HNO_3_, HCl, H_2_O_2_ all purchased from Kemika, Zagreb, Croatia) were used.

The organic matrix of the turmeric samples or reference materials was removed by acidic wet digestion in a closed-vessel microwave assisted digestion system (Berghof Speedwave MWS-2). Five different acid mixtures were tried, whereby the number of different reagents, their kind and concentration alongside their ratios and volumes was changed during the optimization step. The mixtures were the following: A: 3 mL HNO_3_ conc, B: 6 mL HNO_3_ (50:50 *v*/*v*) + 2 mL H_2_O_2_ (1 mol L^−1^), C: 1 mL HNO_3_ conc + 4 mL H_2_O + 2 mL H_2_O_2_ (1 mol L^−1^), D: 6 mL HNO_3_ conc + 2 mL H_2_O_2_ (1 mol L^−1^) and E: 1 mL HNO_3_ conc + 3 mL HCl conc. The temperature program was the same for all mixtures consisting of three steps (time in min/power in W/temperature in °C): 30/500/120; 30/700/170; 30/400/130. Clear digest solutions were filled up t o a final volume of 25.0 mL by adding ultrapure water. In case of remaining solid particles, a filtration step was needed prior to filling up to the target volume. Each sample as well CRM was digested multiple time using, 200 mg to 250 mg (weighed to the nearest 0.1 mg) dried matter each. Blank solutions were prepared in the same way. 

For determining the method validation parameter precision and trueness (expressed as recovery) four different plant-derived standard reference materials underwent the chosen digestion procedure, namely algae (IAEA 392), apple leaves (NIST SRM 1515), peach leaves (NIST SRM1547) and tomato leaves (NIST SRM1573a).

### 3.3. Measurements

The digest solutions (samples, blanks, CRMs) were analyzed by both inductively coupled plasma atomic emission spectrometry (ICP-AES; Prodigy HD; Teledyne Leeman, Hudson, NH, USA) and inductively coupled plasma mass spectrometry (ICP-MS; Agilent 7500cx, Tokyo, Japan). Whilst no further dilution was done prior to the ICP-AES measurements, a 1:10 dilution step using 1% *v*/*v* nitric acid was carried out prior to the ICP-MS measurements. Both analytical methods have been used for similar analytical problems before [1,22], thus the operational conditions needed no further optimization. For completeness, they are given below in Table 1. The quantification was performed via external five-point calibrations based on multi-elemental standards solutions prepared by appropriate dilution from a multielement stock solution (ICP Multielement Standard IV, Merck, Darmstadt, Germany).

### 3.4. Data Evaluation and Statistics

Data were evaluated considering reagent blank, final volume, dilution step and the mass of sample digested, so that the final contents were obtained in mg/kg of all analytes per each dried sample. Based on triplicates measurements mean value and standard deviation were calculated per sample. The limits of detection (LODs) and the limits of quantification (LOQs) were calculated based on 3 σ and 9 σ of the blank solutions, respectively. For finding the best digestion mixtures, the results for all five procedures were compared using dependent sample t-tests. Principal Component Analysis (PCA) was carried out to see the influence of the single analytes alongside their correlation as well as the influence of the individual samples. Data were used with the same unit after standardization to mean zero and standard deviation one, values below LOD (compare Tables S1–S3) were set zero (0.0) to fill the gaps of missing data. Decision-making was based on a level of significance of 95% for all tests. The Microsoft Office Excel, version 2016 (Microsoft Corporation, 2018. *Microsoft Excel*, Available at: https://office.microsoft.com/excel, accessed on 30 October 2022) as well as the R 4.03 (R Core Team (2022). R: A language and environment for statistical computing. R Foundation for Statistical Computing, Vienna, Austria. URL https://www.R-project.org/, accessed on 30 October 2022) were used for data processing.

## 4. Conclusions

Semi-concentrated nitric acid together with hydrogen peroxide is a reagent mixture that can be used for digest plant derived products, e.g., powdered turmeric. The determined elemental composition is similar to literature data, but for both macro and micro elements, deviations are found. The macro elements were found in the g/kg-range in the following decreasing order: K > Mg > Ca > Na for K1 to K3, whilst K4 showed the order K > Ca, Na > Mg. Minor and trace elements are present in the mg/kg range, differences between the samples were found especially for Al, Cr, Cu, Mn, Rb, Sr, and Zn. The mass fractions of three elements are regulated by the WHO, based on the stipulated limits it can be seen that all analyzed samples can be considered harmless regarding As, Cd, and Pb, except for sample K4, which contained more Cd than allowed. It can be speculated based on literature data that K4 with elevated levels of Na, but also of potentially toxic elements, originates from an area with high soil salinity. The significant differences found for macro- as well as micro-elements underline the high variation of accumulated metals and metalloids in plants given due to different influencing parameters, such as the location of growing place covering soil composition, potential pollution of the site, climatic conditions, the year of harvest and the processing of the plant to finally sold product. Due to a lack of information on these parameters, a profound interpretation cannot be performed, but will be considered in the study design of future projects.

## Figures and Tables

**Table 1:** Instrumental conditions of the analytical methods used in the present study.

Parameter	ICP-OES *	ICP-MS **
Instrument	Prodigy High Dispersive ICP-AES (Teledyne Leeman, Hudson, NH, USA)	Agilent 7500cx ICP-MS (Agilent, Tokyo, Japan)
Output power	1100 W	1500 W
Argon flows	Plasma:18 L/min	Plasma:15 L/min
	Auxiliary: 0.8 L/min	Auxiliary: 0.9 L/min
	Nebulizer: 1 L/min	Nebulizer: 0.2 L/min
Collison cell	-------	On/off depending on element
Nebulizer	Pneumatic (glass concentric)	MicroMist
Spray chamber	Glass cyclonic	Scott double pass
Sample flow	1.0 mL/min	0.3 mL/min

* University of Zagreb (Croatia). ** Örebro University (Sweden)

## Data Availability

Data, associated metadata, and calculation tools are available from the corresponding author.

## Supplementary Materials

The following are available online at https://www.mdpi.com/article/10.3390/molecules27238392/s1, Table S1: Results for optimization of digestion method; Table S2: Validation data; Table S3: Results for mass fractions in samples K1 to K4.

## References

[1] Nwankwo C. (2014). Nutritional Composition of Turmeric (*Curcuma longa*) and its Antimicrobial Properties. Int. J. Eng. Res..

[2] Prasad S., Aggarwal B.B., Benzie I.F.F., Wachtel-Galor S. (2011). Turmeric, the Golden Spice: From Traditional Medicine to Modern Medicine. Herbal Medicine: Biomolecular and Clinical Aspects.

[3] Li S.Y., Yuang W., Deng G., Wang P., Yang P., Aggarwal B.B. (2011). Chemical composition and product quality control of turmeric (*Curcuma longa* L.). Pharm. Crops.

[4] Dosoky N.S., Setzer W.N. (2018). Chemical Composition and Biological Activities of Essential Oils of *Curcuma* Species. Nutrients.

[5] Gupta S.C., Sung B., Kim J.H., Prasad S., Li S., Aggarwal B.B. (2013). Multitargeting by turmeric, the golden spice: From kitchen to clinic. Mol. Nutr. Food Res..

[6] Hewlings S.J., Kalman D.S. (2017). Curcumin: A Review of Its Effects on Human Health. Foods.

[7] Verpoort R., Contin A., Memelink J. (2002). Biotechnology for the production of plant secondary metabolites. Phytochem. Rev..

[8] Nasim S.A., Dhir B. (2010). Heavy metals alter the potency of medicinal plants. Rev. Environ. Contam. Toxicol..

[9] World Health Organization (1998). Quality Control Methods for Medicinal Plant Materials.

[10] Shahid M., Dumat C., Khalid S., Schreck E., Xiong T., Niazi N.K. (2017). Foliar heavy metal uptake, toxicity and detoxification in plants: A comparison of foliar and root metal uptake. J. Hazard Mater..

[11] Goyer R.A. (1997). Toxic and essential metal interactions. Annu. Rev. Nutr..

[12] Maghrabi I. (2014). Determination of some mineral and heavy metals in Saudi Arabia popular herbal drugs using modern techniques. Afr. J. Pharm. Pharmacol..

[13] Muralidharan L., Muralidharan C., Gaur S. (2019). Analysis of Heavy and Trace Metals in Golden Ingredient Turmeric (Curcuma longa) of Mumbai, Maharashtra. 10.13140/RG.2.2.25945.88168.

[14] Silva P., Francisconi L., Gonçalves R. (2016). Evaluation of Major and Trace Elements in Medicinal Plants. J. Braz. Chem. Soc..

[15] Zeiner M., Cindrić I.J. (2017). Review–trace determination of potentially toxic elements in (medicinal) plant materials. Anal. Methods.

[16] Lyn J., Ramsey M., Fussell R., Wood R. (2003). Measurement uncertainty from physical sample preparation: Estimation including systematic error. Analyst.

[17] Ramsey M., Rostron P., Ellison S. (2019). Eurachem/EUROLAB/CITAC/Nordtest/AMC Guide: Measurement Uncertainty Arising from Sampling: A Guide to Methods and Approach.

[18] Bendicho C., Lavilla I., Pena-Pereira F.F., Romero V. (2012). Green chemistry in analytical atomic spectrometry: A review. J. Anal. At. Spectrom..

[19] Machado R., Fernandes A.D., Babos D., Castro J., Costa V., Sperança M.A., Garcia J., Gamela R., Filho E. (2019). Solid sampling: Advantages and challenges in atomic spectrometry—A critical review. J. Anal. At. Spectrom..

[20] Engin M., Uyanik A., Cay S., Icbudak H. (2010). Effect of the Adsorptive Character of Filter Papers on the Concentrations Determined in Studies Involving Heavy Metal Ions. Adsorp. Sci. Technol..

[21] May T.W., Wiedmeyer R.H. (1998). A Table of Polyatomic Interferences in ICP-MS. At. Spectrosc..

[22] Zeiner M., Kuhar A., Cindrić I.J. (2019). Geographic Differences in Element Accumulation in Needles of Aleppo Pines (*Pinus halepensis* Mill.) Grown in Mediterranean Region. Molecules.

[23] Zeiner M., Cindrić I.J., Nemet I., Franjković K., Salopek Sondi B. (2022). Influence of Soil Salinity on Selected Element Contents in Different *Brassica* Species. Molecules.

[24] Burger A., Lichtscheidl I. (2019). Strontium in the environment: Review about reactions of plants towards stable and radioactive strontium isotopes. Sci. Total Environ..

[25] Wani A.L., Ara A., Usmani J.A. (2015). Lead toxicity: A review. Interdiscip. Toxicol..

[26] Koons A.L., Rajasurya V. (2022). Cadmium Toxicity. StatPearls.

[27] Andersson C.M. (2022). Determination of Bio-Accessible Amounts of Metal Trace Elements in Baby Food Using In Vitro Artificial Digestion. Bachelor’s Thesis.

