# Supplementary material for: Multielement Determination in Turmeric (Curcuma longa L.) Using Different Digestion Methods
Source: Molecules. 2022 Dec 1;27(23):8392. doi: 10.3390/molecules27238392 (PMC9741154; doi:10.3390/molecules27238392)
Supplement: Supplementary file 1 [file molecules-27-08392-s001.zip › molecules-1906635-supplementary.pdf]

Table S1: Mass fractions (mean value alongside standard deviation (SD), all in mg/kg) for all analytes in sample K1 after digestion using five different digestions mixtures A – E

Analyte	A mean	A SD	B mean	B SD	C mean	C SD	D mean	D SD	E mean	E SD
Ag	<LOD	n/a	1.10E-02	6.00E-03	4.40E-02	3.80E-02	<LOD	n/a	9.00E-03	1.60E-02
Al	3.03E+02	8.80E+01	3.80E+02	8.80E+01	5.06E+02	2.70E+01	1.59E+02	8.00E+01	5.45E+02	1.43E+02
As	6.40E-02	6.30E-02	5.40E-02	1.40E-02	4.10E-02	3.00E-03	4.50E-02	1.00E-02	1.70E-01	1.31E-01
Ba	1.14E+01	1.12E+00	1.27E+01	5.12E-01	1.20E+01	3.76E-01	1.18E+01	4.51E-01	1.22E+01	4.37E-01
Be	3.00E-03	3.40E-03	6.00E-03	1.60E-03	3.00E-03	2.20E-03	1.00E-03	9.00E-04	<LOD	n/a
Bi	<LOD	n/a	3.00E-02	4.00E-02	3.00E-02	5.00E-02	1.00E-01	7.00E-02	3.00E-02	4.00E-02
Ca	1.48E+03	1.38E+02	1.54E+03	1.42E+02	1.53E+03	2.90E+01	1.69E+03	3.58E+02	1.33E+03	1.90E+01
Cd	5.30E-02	3.00E-03	6.20E-02	3.00E-02	9.10E-02	7.50E-02	8.20E-02	6.10E-02	3.80E-02	2.00E-03
Co	5.06E-01	3.50E-02	5.46E-01	1.20E-02	5.82E-01	9.60E-02	5.27E-01	5.00E-03	5.97E-01	2.00E-02
Cr	1.31E+00	1.25E-01	2.16E+00	5.47E-01	1.38E+00	4.19E-01	1.15E+00	2.40E-02	2.55E+00	1.82E-01
Cu	1.44E+01	1.20E-01	7.13E+00	9.20E-01	1.22E+01	1.44E+00	1.75E+01	4.19E+00	1.42E+01	9.10E-01
Fe	1.63E+02	4.58E+01	4.53E+02	7.49E+01	3.81E+02	2.74E+01	1.47E+02	1.22E+01	7.27E+02	5.49E+01
Ga	7.50E-01	5.00E-02	8.40E-01	3.10E-02	8.60E-01	8.70E-02	7.50E-01	4.50E-02	7.60E-01	7.00E-03
K	2.94E+04	1.86E+03	2.77E+04	2.81E+02	3.00E+04	7.62E+02	2.93E+04	1.68E+03	2.19E+04	3.05E+03
Li	1.19E-01	6.00E-03	1.48E-01	1.20E-02	2.27E-01	1.10E-01	8.40E-02	1.70E-02	1.19E-01	1.50E-02
Mg	3.12E+03	2.67E+02	3.06E+03	1.42E+02	3.20E+03	7.50E+01	3.12E+03	1.95E+02	2.60E+03	2.20E+02
Mn	9.12E+01	7.10E+00	9.34E+01	2.20E+00	9.45E+01	3.10E+00	9.30E+01	3.30E+00	8.87E+01	1.60E+00
Mo	1.30E-01	5.40E-02	1.49E-01	1.10E-02	8.80E-02	5.30E-02	1.35E-01	2.50E-02	2.71E-01	8.50E-02
Na	4.15E+02	4.23E+01	4.36E+02	1.66E+01	4.07E+02	1.49E+01	3.87E+02	4.70E+01	3.53E+02	7.50E+00
Ni	1.20E+00	1.79E-01	<LOD	n/a	1.80E+00	1.65E-01	1.56E+00	1.00E-01	1.66E+00	8.95E-02
Pb	5.74E-01	5.70E-02	4.87E-01	2.10E-02	2.24E-01	2.12E-02	<LOD	n/a	3.18E-01	1.27E-01
Rb	4.50E+00	3.60E-01	4.80E+00	1.10E-01	4.70E+00	1.10E-01	4.60E+00	1.20E-01	4.64E+00	1.00E-02
Se	2.54E-01	1.90E-01	7.50E-02	1.50E-01	<LOD	n/a	9.70E-02	1.00E-02	1.60E+00	1.15E+00
Sr	1.61E+01	1.60E+00	1.72E+01	4.50E-01	1.69E+01	2.30E-01	1.64E+01	2.50E-01	1.67E+01	5.90E-01
Te	<LOD	n/a	2.00E-03	2.00E-03	3.00E-03	9.00E-03	2.00E-03	3.00E-03	1.80E-02	2.40E-02
Tl	1.70E-02	3.00E-03	2.10E-02	2.00E-03	7.10E-02	4.40E-02	2.00E-02	4.00E-04	1.30E-02	2.00E-03
V	9.10E-01	2.00E-02	1.31E+00	2.64E-01	1.23E+00	8.80E-02	7.77E-01	9.50E-02	1.13E+00	4.90E-02
Zn	2.62E+01	1.10E+00	2.42E+01	2.60E+00	2.82E+01	2.30E+00	2.39E+01	3.00E+00	2.28E+01	1.70E-02

<LOD ….. below limit of detection; n/a….. not applicable

Table S2: Validation parameter for the analytes in alphabetical order (R^2, coefficients a and b of linear trendlines y = a x + b with x being the molar concentration in the digest solution; recovery as mean of different CRMs where applicable; RSD per sample)

Analyte	R^2	a	b	recovery in %	RSD in %	LOD in mg/kg
Ag	0.999952917	0.404627966	-9.83056E-05	107	3.9	0.0042
Al	0.999989578	0.348731518	-0.001430122	82	1.3	0.017
As	0.999975637	0.023224992	-0.000144855	98	3.6	0.00061
Ba	0.999929279	0.122668449	9.93035E-05	86	1.3	0.0034
Be	0.999999914	0.043985658	-0.000119198	Not certified	3.9	0.00094
Bi	0.998814113	0.617889276	-0.004646787	Not certified	3.7	0.0098
Ca	0.999993326	0.000939427	0.00017514	113	1.5	0.15
Cd	0.999999964	0.07538205	-5.95123E-05	86	1.5	0.0007
Co	0.999995173	0.657015064	-0.000198107	84	1.4	0.0042
Cr	0.99999823	0.020380214	0.000102398	115	3.6	0.0015
Cu	0.999977913	0.269458161	0.002661113	89	0.7	0.0084
Fe	0.999995767	0.141687078	-0.002047974	109	0.6	0.061
Ga	0.999982501	0.494735691	0.000356131	Not certified	0.6	0.0068
K	0.999995302	0.005732367	0.004628044	93	1.3	1.9
Li	0.999999088	0.12061962	-0.000182307	82	2.2	0.016
Mg	0.999994222	0.253373116	-0.000320669	102	1.2	0.12
Mn	0.999997389	0.722470822	-0.000416169	84	1.1	0.0032
Mo	0.999937689	0.140071876	-0.000336881	113	3.8	0.0072
Na	0.99997991	0.367264706	0.139843544	103	0.5	1.9
Ni	0.999984065	0.151314115	-0.002142909	111	1.4	0.0024
Pb	0.999928323	1.017773186	0.000146428	89	1.5	0.0016
Rb	0.999978782	0.589798864	-0.000379423	95	1.3	0.0022
Se	0.999999601	0.000740481	-0.000192492	115	4.0	0.049
Sr	0.999983148	0.799827863	5.97452E-05	90	1.1	0.0036
Te	0.999999857	0.012589354	1.98354E-05	Not certified	2.6	0.00012
Tl	0.999940616	0.735614877	0.000220781	Not certified	2.0	0.0035
U	0.999997293	1.039423869	1.93696E-05	111	1.0	0.00079
V	0.999997952	0.122708256	-0.000143079	104	2.2	0.0041
Zn	0.999992887	0.089080162	0.001927291	114	1.4	0.011

Table S3: Mass fractions (mean value alongside standard deviation (SD), all in mg/kg) for all analytes in samples K1 – K4 after digestion using five different digestions mixtures B

Analyte	K1 mean	K1 SD	K2 mean	K2 SD	K3 mean	K3 SD	K4 mean	K4 SD
Ag	<LOD	n/a	2.40E-02	3.00E-03	9.00E-03	5.12E-03	5.70E+00	7.10E+00
Al	3.79E+02	8.80E+01	2.68E+02	3.87E+01	7.30E+01	1.90E+01	<LOD	n/a
As	7.50E-02	1.40E-02	4.80E-02	2.10E-02	2.80E-02	1.90E-02	1.72E-01	3.80E-02
Ba	1.20E+01	5.12E-01	5.69E+00	2.18E-01	8.99E+00	8.04E-01	1.31E+01	6.06E-01
Be	2.00E-03	1.60E-03	2.00E-03	1.00E-03	1.00E-03	1.00E-03	<LOD	n/a
Bi	4.00E-02	3.60E-03	1.00E-02	7.00E-03	9.50E-03	1.35E-02	2.00E-01	1.80E-01
Ca	1.52E+03	1.42E+02	1.41E+03	9.61E+02	1.20E+03	1.48E+02	1.29E+04	1.33E+03
Cd	6.50E-02	3.00E-02	6.40E-02	1.70E-02	3.30E-02	4.00E-03	4.35E-01	3.98E-02
Co	5.52E-01	1.20E-02	4.04E-01	1.65E-01	2.90E-01	4.70E-02	7.37E-01	8.57E-02
Cr	1.71E+00	5.47E-01	6.64E-01	6.84E-01	8.34E-01	1.73E-01	5.00E+00	4.40E+00
Cu	1.31E+01	9.00E-01	6.22E+00	1.10E+00	<LOD	n/a	3.27E+00	1.30E+00
Fe	3.74E+02	7.49E+01	2.36E+02	9.91E+01	1.92E+02	1.60E+01	5.42E+02	5.45E+01
Ga	7.90E-01	3.12E-02	3.80E-01	2.36E-02	5.70E-01	6.00E-02	1.14E+00	5.60E-01
K	2.77E+04	2.81E+02	2.63E+04	1.77E+03	3.35E+04	2.13E+03	3.61E+04	1.73E+03
Li	1.39E-01	1.20E-02	4.20E-02	1.60E-02	5.50E-02	3.00E-02	4.67E-01	2.71E-01
Mg	3.02E+03	1.42E+02	2.73E+03	1.51E+02	2.29E+03	2.44E+02	3.20E+03	8.30E+02
Mn	9.22E+01	2.20E+00	1.85E+02	2.55E+00	5.91E+01	6.40E+00	7.63E+01	5.37E+01
Mo	1.55E-01	1.10E-02	3.48E+00	4.68E+00	9.00E-02	1.40E-02	2.27E+00	4.48E-01
Na	4.00E+02	1.66E+01	2.23E+02	6.60E+01	4.08E+02	3.46E+01	1.31E+04	1.75E+03
Ni	1.21E+00	1.56E-01	9.06E-01	1.28E-01	<LOD	n/a	5.08E-01	4.23E-01
Pb	2.83E-01	2.10E-02	2.99E-01	1.10E-01	4.20E-01	8.20E-02	1.44E+00	1.90E-01
Rb	4.60E+00	1.09E-01	6.00E+00	1.07E-01	6.20E+00	5.00E-01	8.60E+00	2.20E+00
Se	3.89E-01	1.51E-01	9.70E-02	1.22E-01	6.00E-02	5.40E-03	<LOD	n/a
Sr	1.66E+01	4.50E-01	8.60E+00	1.98E-01	1.00E+01	7.00E-01	4.10E+01	2.44E+01
Te	4.00E-03	2.00E-03	2.00E-03	2.00E-03	1.00E-02	1.00E-02	9.00E-03	1.00E-02
Tl	2.80E-02	2.00E-03	1.80E-02	3.00E-03	7.00E-03	1.20E-03	2.96E-01	4.00E-01
V	1.07E+00	2.64E-01	9.62E-01	5.09E-01	8.07E-01	5.40E-02	1.04E+00	1.26E+00
Zn	2.51E+01	1.70E-02	3.97E+01	3.50E+00	8.00E+00	1.00E+00	2.84E+01	1.83E+01
